# Supplementary material for: TXNIP Promotes Human NK Cell Development but Is Dispensable for NK Cell Functionality
Source: Int J Mol Sci. 2022 Sep 26;23(19):11345. doi: 10.3390/ijms231911345 (PMC9570291; doi:10.3390/ijms231911345)
Supplement: Supplementary file 1 [file ijms-23-11345-s001.zip › ijms-1876191-supplementary.pdf]

Supplementary Table S1. Utilized antibodies for flow cytometry

Marker	Alternative name	Fluorochrome	Clone	Supplier
CD3	CD3	APC	SK7	BD Biosciences, San Jose, CA, USA
CD11a	LFA-1	PE, Alexa Fluor 700	HI111	Biolegend, San Diego, CA, USA
CD14	CD14	APC	REA599	Miltenyi Biotec, Leiden, The Netherlands
CD16	FcγRIIIA	PE, APC	B73.1	Biolegend, San Diego, CA, USA
CD19	CD19	APC	SJ25C1	Thermo Fisher Scientific, Waltham, MA, USA
CD34	CD34	PE, Pacific Blue	AC136	Miltenyi Biotec, Leiden, The Netherlands
CD45	CD45	APC-Fire™750	2D1	Biolegend, San Diego, CA, USA
CD45RA	CD45RA	APC, Brilliant Violet 421	HI100	Biolegend, San Diego, CA, USA
CD56	NCAM1	VioBlue, APC	5.1H11	Biolegend, San Diego, CA, USA
CD94	KLRD1	PerCP-Cy5.5	DX22	Biolegend, San Diego, CA, USA
CD107a	LAMP1	APC	H4A3	BD Biosciences, San Jose, CA, USA
CD117	KIT	PECy7	104D2	Thermo Fisher Scientific, Waltham, MA, USA
CD122	IL2RB	PE	MIKβ3	BD Biosciences, San Jose, CA, USA
CD158a,h	KIR2DL1/KIR2DS1	PE	REA1010	Miltenyi Biotec, Leiden, The Netherlands
CD158b1/b2	KIR2DL2/KIR2DL3	PE	REA1006	Miltenyi Biotec, Leiden, The Netherlands
CD158e1/e2	KIR3DL1/KIR3DS1	PE	REA168	Miltenyi Biotec, Leiden, The Netherlands
CD158i	KIR4DS1	PE	REA860	Miltenyi Biotec, Leiden, The Netherlands
NKG2A	CD159a	APC	REA110	Miltenyi Biotec, Leiden, The Netherlands
NKG2C	CD159c	PE	FAB138P	R&D systems, Minneapolis MN, USA
CD226	DNAM-1	Alexa Fluor 647	DX11	BD Biosciences, San Jose, CA, USA
NKG2D	CD314	APC	1D111	Biolegend, San Diego, CA, USA
NKp46	CD335;NCR1	PECy7	9E2	Biolegend, San Diego, CA, USA
NKp44	CD336;NCR2	eFluor450, APC	44.189	Thermo Fisher Scientific, Waltham, MA, USA
NKp30	CD337	PE	P30-15	Biolegend, San Diego, CA, USA
Granzyme B	Granzyme B	PE	GB11	Thermo Fisher Scientific, Waltham, MA, USA
HLA-DR	HLA-DR	eFluor780	LN3	Thermo Fisher Scientific, Waltham, MA, USA
Perforin	Perforin	PE	dG9	Thermo Fisher Scientific, Waltham, MA, USA
AIOLOS	IKZF3	PE	16D9C97	Biolegend, San Diego, CA, USA
EOMES	EOMES	PE, eFluor660	WD1928	Thermo Fisher Scientific, Waltham, MA, USA
HELIOS	IKZF2	APC	22F6	Biolegend, San Diego, CA, USA
HOBIT	ZNF683	Alexa Fluor 647	Sanquin-Hobit/1	BD Biosciences, San Jose, CA, USA
IFN-γ	IFN-γ	eFluo 660	4S.B3	Thermo Fisher Scientific, Waltham, MA, USA
IKZF2	HELIOS	APC	22F6	Biolegend, San Diego, CA, USA
IRF8	IRF8	APC	V3GYWCH	Thermo Fisher Scientific, Waltham, MA, USA
NFIL3	E4BP4	PE	MABA223	Thermo Fisher Scientific, Waltham, MA, USA
PRDM1	Blimp-1	PE	6D3	BD Biosciences, San Jose, CA, USA
RUNX2	AML3, CBFA1	PE	D1L7F	Cell Signalling Technologies, Leiden, The Netherlands
RUNX3	AML42 CBFA3	PE	R3-5G4	BD Biosciences, San Jose, CA, USA
TBET	TBET	PE	4B10	Thermo Fisher Scientific, Waltham, MA, USA
TNF-α	TNF-α	PECy7	Mab11	Thermo Fisher Scientific, Waltham, MA, USA
TOX	TOX	PE	TXRX10	Thermo Fisher Scientific, Waltham, MA, USA

Allophycocyanin (APC), Peridinin Chlorophyll Protein-Cyanin5.5 (PerCP-Cy5.5), Phycoerythrin (PE), Phycoerythrin-Cyanin7 (PECy7)

Supplementary Table S2. qPCR primers

Gene	Sense	Sequence
TXNIP	forward	5'-ATATGGGTGTGTAGACTACTGGG-3'
	reverse	5'-GACATCCACCAGATCCACTACT-3'
TXNIP (flanking shRNA binding sites)	forward	5'-GGTGTGTAGACTACTGGGTGA-3'
	reverse	5'-AGACACAGGTGCCATTAAATCAG-3'
BTG3	forward	5'-ATGAAATTGCTGCCGTTGTCT-3'
	reverse	5'-GCCTGTCCTTTCGATGGTTTT-3'
CDC42	forward	5'-CCATCGGAATATGTACCGACTG-3'
	reverse	5'-CTCAGCGGTCGTAATCTGTCA-3'
CDKN1A	forward	5'-AGGTGGACCTGGAGACTCTCAG-3'
	reverse	5'-TCCTCTTGGAGAAGATCAGCCG-3'
LTV1	forward	5'-GAAAGCTGTGTCTTTTCACTTGG-3'
	reverse	5'-GCACTGAAGGTACTTGAGGGA-3'
RRP15	forward	5'-ATGGTAACTGGAGCCGTAGCGT -3'
	reverse	5'-AGCATCACCCTCACTGTCAGCT-3'
GAPDH	forward	5'-TCCTCTGACTTCAACAGCGACA-3'
	reverse	5'-GTGGTCGTTGAGGGCAATG-3'
TBP	forward	5'-CACGAACCACGGCACTGATT-3'
	reverse	5'-TTTTCTTGCTGCCAGTCTGGAC-3'
YWHAZ	forward	5'-ACTTTTGGTACATTGTGGCTTCAA-3'
	reverse	5'-CCGCCAGGACAAACCAGTAT-3'

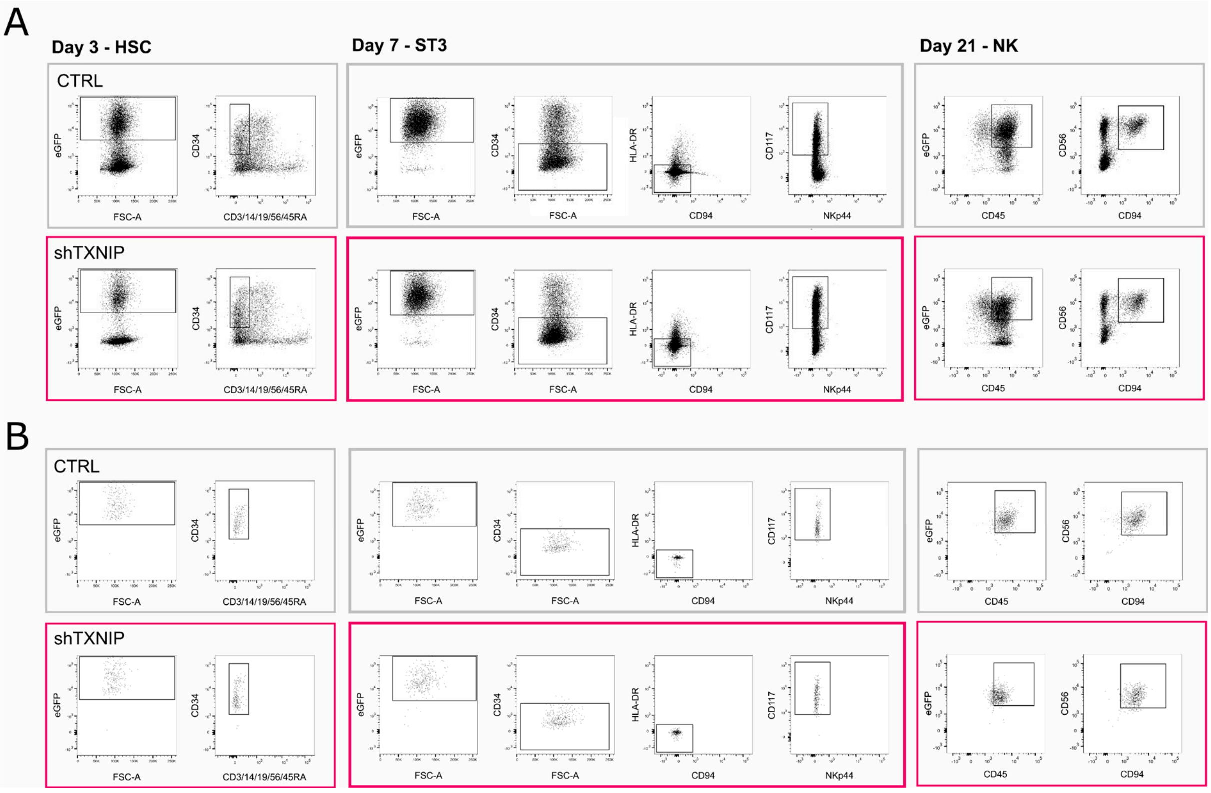

Supplemental Figure S1. Gating strategy and purity of sorted populations. Flow cytometric gating strategy of d3 HSC, d7 ST3 and d21 NK cells pre- (A) and post-sort (B). Cells were previously gated on the viable population based on propidium iodide-negativity and on lymphocytes based on FSC and SSC
